# Characterization of Root and Foliar-Applied Iron Oxide Nanoparticles (α-Fe_2_O_3_, γ-Fe_2_O_3_, Fe_3_O_4_, and Bulk Fe_3_O_4_) in Improving Maize (*Zea mays* L.) Performance

**DOI:** 10.3390/nano13233036

**Published:** 2023-11-28

**Authors:** Nauman Yousaf, Muhammad Ishfaq, Hassan Ali Qureshi, Atif Saleem, Haofeng Yang, Muhammad Fahad Sardar, Chunqin Zou

**Affiliations:** 1 State Key Laboratory of Nutrient Use and Management, College of Resources and Environmental Sciences, China Agricultural University, Beijing 100193, China; mahr.nauman@cau.edu.cn (N.Y.); ishfaq@cau.edu.cn (M.I.); s20223030466@cau.edu.cn (H.Y.); 2 College of Life Sciences and Oceanography, Shenzhen University, Shenzhen 518061, China; 3 Department of Mechanical and Materials Engineering, University of Turku, FI-20014 Turku, Finland; 4 Frontiers Science Centre for Flexible Electronics, Institute of Biomedical Materials and Engineering, Northwestern Polytechnical University, Xi’an 710072, China; atif@nwpu.edu.cn; 5 Key Laboratory of Ecological Prewarning, Protection and Restoration of Bohai Sea, Ministry of Natural Resources, School of Life Sciences, Shandong University, Qingdao 266237, China; fahadsardar@sdu.edu.cn

**Keywords:** nanoparticles, Fe oxide, plant growth, Fe nutrition, oxidative stress, root and foliar application, maize

## Abstract

Iron (Fe) oxide nanoparticles (NPs) improve crop growth. However, the comparative effect of root and foliar-applied different sources of Fe oxide NPs on plant performance at morphological and physiological levels still needs to be discovered. In this study, we characterized the growth and physiological responses of hydroponic-cultured maize seedlings to four sources of Fe (i.e., α-Fe_2_O_3_, γ-Fe_2_O_3_, Fe_3_O_4_ NPs, and bulk Fe_3_O_4_) and two application methods (root vs. foliar). Results showed that Fe concentration in root and shoot increased by elevating the level of NPs from 100 mg L^−1^ to 500 mg L^−1^. Overall, the responses of maize seedlings to different sources of Fe oxide NPs were as follows: Fe_3_O_4_ > γ-Fe_2_O_3_ > α-Fe_2_O_3_ > bulk Fe_3_O_4_. The application of Fe at concentrations ranging from 100 mg L^−1^ to 500 mg L^−1^ had no significant effects on various growth parameters of maize, including biomass, chlorophyll content, and root length. Iron oxide NPs increased the plant biomass by 23–37% by root application, whereas it was 5–9% by foliar application. Chlorophyll contents were increased by 29–34% and 18–22% by foliar and root applications, respectively. The non-significant response of reactive oxygen species (i.e., superoxide dismutase, catalase, and peroxidase) suggested optimum maize performance for supplementing Fe oxide NPs. A confocal laser scanning microscope suggested that Fe oxide NPs entered through the epidermis and from the cortex to the endodermis. Our results provide a scientific basis that the root application of Fe_3_O_4_ at the rate of 100 mg L^−1^ is a promising approach to obtain higher maize performance and reduce the quantity of fertilizer used in agriculture to minimize environmental effects while improving crop productivity and quality. These findings demonstrated the tremendous potential of Fe NPs as an environmentally friendly and sustainable crop approach.

## 1. Introduction

Iron (Fe), an essential mineral nutrient, is the third most deficient micronutrient in plants [1]. It performs a wide array of physiological and biochemical processes, i.e., photosynthesis, respiration, DNA synthesis, nitrate synthesis, and nitrogen fixation in plants [1,2]. The structure and function of photosynthetic apparatus can be disturbed by Fe deficiency that degrades the chloroplast and reduces the chlorophyll in plants [3]. Additionally, Fe deficiency induces morphological changes in roots, like swelling of root tips and formation of lateral roots and root hairs [4]. Iron availability is scarce at calcareous and alkaline pH, which reduces plant growth, yield, and quality of fruit [5,6]. Iron fertilizer (i.e., FeSO_4_, EDTA-Fe) are widely used in improving the Fe nutrition of plants [7]. Foliar-applied FeSO_4_ positively affects the nutritional composition and herbage production of teosinte [8,9]. However, they have some negative impacts, i.e., environmental pollution, FeSO_4_ is readily water soluble and can be leached down, or Fe^2+^ rapidly converted into plant unavailable Fe^3+^, whereas EDTA-Fe has the chelating ability with other metals that can enhance their availability to plants [10,11]. EDTA reduces the plant biomass but is effective in phytoremediation [12].

Nanotechnology is experiencing a growing trend in plant sciences, biomedicines, and environmental remediation due to its specific physicochemical properties and surface area [13]. The ferromagnetic characteristics of iron oxide nanoparticles (NPs) such as magnetite (Fe_3_O_4_), maghemite (γ-Fe_2_O_3_), and hematite (α-Fe_2_O_3_) remain a topic of interest for future studies [14]. Bulk magnetic components contain regions called magnetic domains, where magnetic moments are aligned and are categorized based on their interactions and impact on the material’s reactions to magnetic fields under various temperatures [15]. Magnetotactic bacteria interestingly have magnetic behavior; inside of these bacteria are magnetic nanoparticles that are called as magnetosomes [16]. Magnetic properties of Fe NPs are dependent on particle size [17]. The size and shape of NPs play a very vital role in stem cell therapy as well as in the field of nanomedicine [18]. Thus, due to their high surface area and small size, iron oxide NPs based fertilizers perform better in terms of crop growth and yield [19].

Iron dynamic in soil-plant systems is well documented, and the uptake and translocation of Fe-based NPs vary widely with shape, size, concentration, and plant species [20,21]. The size of cell wall pore is much smaller (3.5–5 nm) than the size of most of the NPs [22], and NPs can enter the plants through different pathways such as aquaporin membrane transport system [23]. After transportation through symplast and apoplast pathways, NPs translocate and accumulate in the plant cells by xylem and phloem [24,25,26]. Finally, vascular tissues play a significant role in the long-distance transportation of NPs [27,28].

The application of γ-Fe_2_O_3_ NPs increases the root length, biomass, plant height, and chlorophyll contents of peanuts (*Arachis hypogaea*) [29]. Ref. [30] found that soybean (*Glycine max* L. Oxley) takes up Fe_3_O_4_ NPs, translocates it from root-to-shoot tissues, and improves chlorophyll contents and photosynthetic activity. Fe_2_O_3_ NPs application with a concentration of 200–400 mg L^−1^ and size range of 10–50 nm can reduce arsenic toxicity and improve the growth of mung bean [31]. Similarly, 1000 mg L^−1^ α-Fe_2_O_3_ (hematite) application improves peanut growth [29]. Foliar spray of Fe-based NPs (i.e., Fe_3_O_4_) increases chlorophyll content, photosynthesis and the biomass of maize seedlings [32]. Vibrating sample magnetometry (VSM) analysis indicated that magnetite (Fe_3_O_4_) and maghemite (γ-Fe_2_O_3_) with 50, 100, 200 mg L^−1^ increase Fe translocation and content in barley seedlings [33]. Hydroponically grown pumpkin (*Cucurbita mixta*) shows higher root-shoot Fe_3_O_4_ NPs accumulation. These studies suggest that Fe NPs-based fertilizers can be promising in improving crop growth and agricultural productivity. However, the effect of Fe NPs can vary across different crop groups, NPs sources and application methods [34]. Thus, the translocation mechanism, physiological changes, and antioxidant enzymes regulated by Fe oxide NPs by different NPs sources and application methods (i.e., soil vs. foliar) require in-depth investigations.

Global maize production has increased in the past few decades, and maize is the leading cereal crop by production of 5.8 tons per ha on 197 million ha land worldwide [35]. However, Fe content is significantly decreased in cereals due to historically low Fe fertilizer inputs and rising climate change-related concerns [2,7,9]. Foliar application of Fe fertilizer increases the maize grain yield [9,36]. Nitrogen fertilization coupled with Fe foliar spray increases not only the photosynthetic rate but also the yield of maize [37,38]. This study was designed to provide deeper insight into different sources of Fe oxide NPs (α-Fe_2_O_3_, γ-Fe_2_O_3_, Fe_3_O_4_) and application methods (root vs. foliar) in improving the performance of maize seedlings. Given different physiological characteristics, i.e., chlorophyll content, antioxidant enzyme activity, and Fe contents in root-shoot tissues, our findings will provide a scientific basis to better evaluate the source and application method of Fe oxide NPs in improving maize performance and agricultural productivity.

## 2. Materials and Methods

### 2.1. Characterization of Fe Oxide NPs

Non-stoichiometric Iron oxide NPs (α-Fe_2_O_3_, γ-Fe_2_O_3_, and Fe_3_O_4_) and bulk Fe_3_O_4_ with sizes ranging from 10–30 nm and 142 nm, respectively, were procured from Macklin Inc. (Shanghai, China). The NPs were characterized based on their size and shape by using X-ray diffraction (XRD), transmitting electron microscopy (TEM), and selected area electron microscopy (SAED).

#### 2.1.1. X-ray Diffraction

A dried sample of NPs was placed on a sample holder and passed through X-rays, thereby obtaining a diffraction pattern. The obtained diffraction pattern was used to measure the crystalline structure.

#### 2.1.2. Transmitting Electron Microscope

A TEM was used to determine the morphology, size, and structure of NPs. It comprises different steps, sample preparation, imaging, and analysis.

#### 2.1.3. Selected Area Electron Microscopy

A thin crystal sample was illuminated by beam of electrons. Under paralleled electron irradiation, an SAED pattern was obtained, and a particular aperture in the image’s plane was then used to evaluate only a specific sample region.

### 2.2. Experimental Setup and Plant Growth Conditions

Maize (*Zee mays* L. cv. Zhengdan 958) seeds were immersed in 10% H_2_O_2_ for 30 min and then washed with deionized water at least thrice. Seeds were spread on the petri plates with two layers of filter paper to keep the moisture. After 48 h, seedlings with 1 cm primary root length were wrapped using filter paper and grown in a standard growth chamber. After one week, consistent and uniform-sized seedlings were transferred into Hoagland’s nutrient solution with macro- and micronutrients as follows: 0.5 mmol L^−1^ Ca(NO_3_)_2_, 0.1875 mmol L^−1^ K_2_SO_4_, 0.025 mmol L^−1^ KCl, 0.0625 mmol L^−1^ KH_2_PO_4_, 0.1625 mmol L^−1^ MgSO_4_·7H_2_O, 0.25 × 10^−3^ μmol L^−1^ H_3_BO_3_, 0.25 × 10^−3^ μmol L^−1^ MnSO_4_·H_2_O, 0.25 × 10^−3^ μmol L^−1^ ZnSO_4_·7H_2_O, 0.25 × 10^−4^ μmol L^−1^ CuSO_4_·5H_2_O, and 1.25 × 10^−6^ μmol L^−1^ (NH_4_)6Mo_7_O_24_. The pH of the nutrient solution was maintained at around 6.8 with 1 mol L^−1^ NaOH. In case of root application treatment, the 100 mg L^−1^ and 500 mg L^−1^ of α-Fe_2_O_3_, γ-Fe_2_O_3_, Fe_3_O_4_ NPs, and bulk Fe_3_O_4_ (recorded as α-Fe_2_O_3_-1, γ-Fe_2_O_3_-1, Fe_3_O_4_-1, and bulk Fe_3_O_4_-1 and α-Fe_2_O_3_-5, γ-Fe_2_O_3_-5, Fe_3_O_4_-5, and bulk Fe_3_O_4_-5, respectively) were applied in nutrient solution after one week of acclimation, and later no Fe was supplemented in the control treatment. In this study, foliar spray treatments were conducted using NPs at two stages: once when the plant had four leaves and again when it had six leaves. The NPs were applied at the same concentration as in the root (100 mg L^−1^ and 500 mg L^−1^). No additional source of Fe was supplemented to the Hoagland nutrient solution. Amount of Fe 100 mg L^−1^ and 500 mg L^−1^ was calculated from different sources of Fe NPs. The given concentrations of NPs for root and foliar applications were selected based on previous studies [39,40,41]. To avoid the conglomeration of NPs, NPs suspension was sonicated in the sonic water bath for 30 min (Power sonic 410, Hwashin Technology, Yeongcheon-si, Republic of Korea). Each treatment received three replications. Relative humidity was 60–70%, and the temperature was maintained between 23 °C and 26 °C. The maize seedlings were harvested after 3 weeks of treatment application.

### 2.3. Antioxidant Enzymes Activities

The activities of superoxide dismutase (SOD), catalase (CAT), and peroxidase (POD) were determined in the root and shoot tissues. Harvested root and shoot tissues were rinsed with distilled water to remove the surface contamination. Grinding was done in liquid N_2_ with a pestle and mortar to make fine powder for further analysis. In brief, to extract crude enzymes, shoot (0.3 g) and root (0.4 g) were homogenized separately in 10 mL of 0.05 M pre-cooled phosphate buffer (pH 7.8). After centrifuging the mixture at 4000 rpm for 20 min, it was stored at 4 °C for further analysis. Analysis kits were procured from SolarBio^®^, Beijing, China.

#### 2.3.1. Superoxide Dismutase Activity (SOD) Assay

The reaction mixture consists of 0.5 mL supernatant, 0.5 mL of 130 mmol L^−1^ methionine, 0.5 mL of 750 μmol L^−1^ NBT, 0.5 mL of 100 μmol L^−1^ EDTA-Na, 0.5 mL of 20 μmol L^−1^ lactochrome, and 3.5 mL of 0.05 mol L^−1^ phosphate buffer (pH 7.8). The entirely mixed sample was placed in light for 20 min, and distilled water was used to prepare the control sample and was placed in the dark. Finally, the absorbance was measured at 560 nm [41,42].

#### 2.3.2. Peroxide Activity (POD) Assay

The oxidative process of guaiacol, catalyzed by peroxidase, was used to estimate POD activity [43]. To prepare a 3 mL assay combo, 0.5 mL of crude extract, 28 μL of 0.05 mol L^−1^ guaiacol, and 19 μL of 30% H_2_O_2_ were blended in 100 mmol L^−1^ phosphate buffer (pH 7.0). The prepared samples were run at 470 nm (A470) for every 30 s, and POD activity was calculated regarding absorbance change per minute (A470/min/g FW).

#### 2.3.3. Catalase Activity (CAT) Assay

The CAT activity was calculated by mixing 0.5 mL of supernatant with 3 mL of 100 mmol L^−1^ phosphate buffer (pH 7.0) containing 0.01% H_2_O_2_. CAT activity was measured at 240 nm for 4.5 min after every 30 s [44].

### 2.4. Chlorophyll Contents Assay

Chlorophyll contents were determined with a UV-752 N spectrophotometer (Shanghai Precision Scientific Instrument Co., Ltd., Shanghai, China) using 80% acetone extracts as described [45]. Fresh young leaves (0.5 g) of maize were ground in 10 mL of 80% acetone and centrifuged at 5000 rpm for 5 min to obtain the homogenate. After centrifugation, the supernatant was taken to calculate absorbance at 645 and 663 nm wavelengths, and 80% acetone was used as a blank for reference. Total chlorophyll contents were calculated by following the given formula [46]

Chl a = 12.72 A_663_ − 2.59 A_645_ × mL of acetone/weight of the sample

Chl b = 22.88 A_645_ − 4.67 A_663_ × mL of acetone/weight of the sample

Total chlorophyll contents = Chl a + Chl b

### 2.5. Fe Oxide NPs in Root and Shoot Analysis by Confocal Microscopy

During harvesting, fresh plant samples (leaves and roots) were collected for confocal microscopy LSM 800. Samples for confocal microscopy CLSM 800 imaging were prepared following a standard procedure described earlier [47]. A confocal laser scanning microscope was used to process the images of fluorescent Fe NPs translocation in maize leaves and root samples. Translocation of Fe NPs in maize was determined by the fluorescence imparted by Fluorescein Isothiocyanate (FITC). CLSM images were determined at the 480–495 nm and 550–560 nm. Images were taken under Leica 20 oil immersed lens. For GFP and NR excitation, an Argon laser was used.

### 2.6. Fe Concentration in Maize Plants

After harvesting, the shoot and root tissues of maize seedlings were completely dipped in 0.01 M HNO_3_ to remove the adsorbed NPs on the surface [48]. Then, samples were completely rinsed a couple of times with distilled water and dried for 48 h at 65 °C in an oven. This was followed by 6 mL of concentrated HNO_3_ poured to digest the 0.3 g of the oven-dried samples. The next day, 2 mL of 30% H_2_O_2_ was added to the solution. Samples were digested for 10 min at 120 °C and then for 15 min at 180 °C in a microwave oven (MILESTONE Ethos D) controlled by a time and temperature regulator to get a clear solution. The Fe concentration in digested solution was determined using an inductively coupled plasma-optical emission spectrometer (OPTIMA 3300 DV, Perkin-Elmer, Waltham, MA, USA). The standard plant material (IPE568, Wageningen University, Wageningen, The Netherlands) was used for quality control of the Fe analysis.

### 2.7. Statistical Analysis

Each treatment contained three biological replicates, and the results are presented as mean ± SD (standard deviation). The statistical analysis of all collected data was carried out using three factor factorial ANOVA under CRD followed by Tukey’s HSD test (*p* ≤ 0.05) in the statistical package IBM SPSS 22.

## 3. Results and Discussion

### 3.1. Characterization of Fe NPs by TEM and XRD

**Figure 1:**
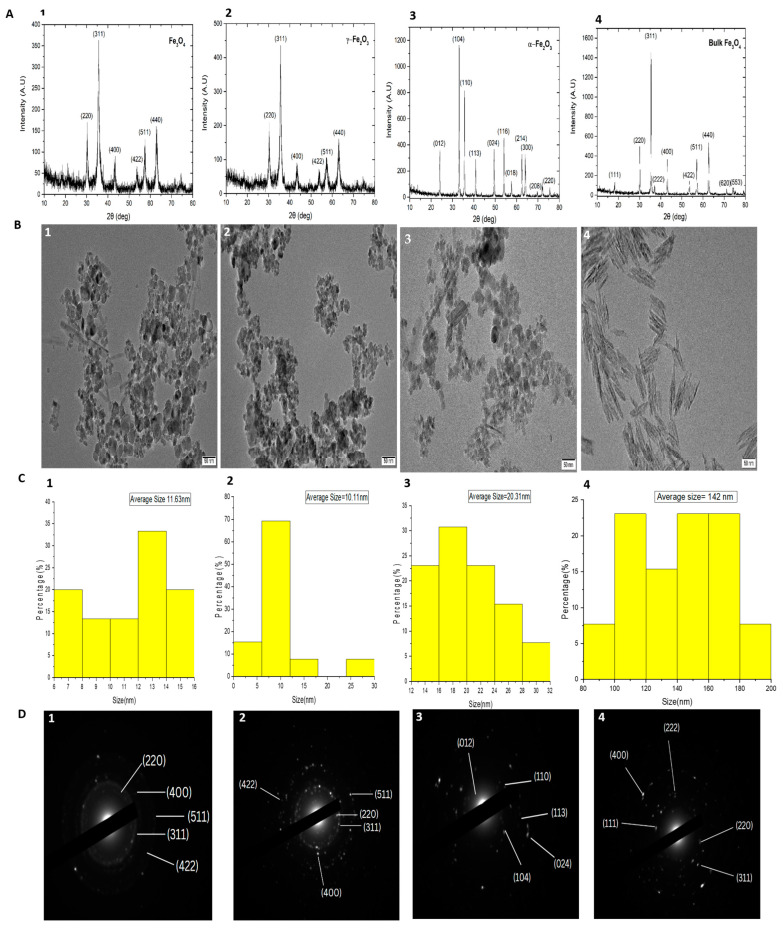
XRD, TEM, Histogram and SAED pattern for structural and size distribution pattern of Fe-NPs. XRD: (**A1**) Fe_3_O_4_, (**A2**) γ-Fe_2_O_3_, (**A3**) α-Fe_2_O_3_, (**A4**) bulk Fe_3_O_4_. TEM: (**B1**) Fe_3_O_4_, (**B2**) γ-Fe_2_O_3_, (**B3**) α-Fe_2_O_3_, (**B4**) bulk Fe_3_O_4_. Histogram: (**C1**) Fe_3_O_4_, (**C2**) γ-Fe_2_O_3_, (**C3**) α-Fe_2_O_3_, (**C4**) bulk Fe_3_O_4_. SAED pattern; (**D1**) Fe_3_O_4_, (**D2**) γ-Fe_2_O_3_, (**D3**) α-Fe_2_O_3_, (**D4**) bulk Fe_3_O_4_.

XRD and TEM were used to characterize the crystalline structure of the tested Fe oxide NPs. The sharp peaks confirmed the crystal structure of α-Fe_2_O_3_, γ-Fe_2_O_3_, Fe_3_O_4_ NPs, and bulk Fe_3_O_4_ (Figure 1). The highest peak was 104 in α-Fe_2_O_3_ and 311 was in γ-Fe_2_O_3_, Fe_3_O_4_ NPs, and bulk Fe_3_O_4_. TEM indicated the mean size of selected NPs ranges 10–30 nm with rounded shape and bulk Fe_3_O_4_ mean size 142 nm with needle shape structure, and SAED was used for the diffraction pattern of spotty bright rings of different intensities represented the purity of the NPs.

### 3.2. Effect of Fe Oxide NPs on Plant Growth and Biomass

The effects of root and foliar application of Fe oxide NPs (α-Fe_2_O_3_, γ-Fe_2_O_3_, Fe_3_O_4_, and bulk Fe_3_O_4_) on the growth parameters (shoot height, root length, and root and shoot dry weight) were analyzed. Results showed that the Fe oxide NPs significantly affected the shoot dry weight, shoot height, root dry weight, and root length (Table 1). Interestingly, shoot height was higher with the root application than in the foliar application of NPs. In the case of root application, compared with the control treatment, the shoot height increased significantly (*p* < 0.05) and it was 87.6 cm, 85.8 cm, and 86.0 cm with Fe_3_O_4_, γ-Fe_2_O_3_, and α-Fe_2_O_3_ application, respectively. Moreover, root application of Fe oxide NPs significantly (*p* < 0.05) affected the root length and dry weight, and Fe_3_O_4_ NPs application increased 47% root length and 52% root dry weight, compared with the control treatment (Table 1). However, we did not find a significant effect in root length and dry weight with foliar application of all Fe oxide NPs. The maximum increase in root dry weight was 31%, and the increase in root length was 8% as compared to the control by foliar application. Overall, foliar application also has a positive effect on maize growth (Table 1). There are different effects on shoot and root growth among different Fe oxide NPs. α-Fe_2_O_3_ increased the plant biomass higher than control and bulk Fe_3_O_4_, and this improvement was lower than γ-Fe_2_O_3_ and Fe_3_O_4_. Bulk Fe_3_O_4_ affected the growth lower than other treatments of Fe oxide NPs by root and foliar application. Additionally, increasing the concentration of NPs from 100 mg L^−1^ to 500 mgL^−1^ did not improve the plant growth and biomass significantly (Table 2). Root morphology was improved with root-applied NPs more than with Foliar applied (Figure S1). The plant biomass was relatively higher in the root application of NPs than in the foliar application. Roots are the primary tissues of plants that NPs can contact and increase the exposure time with NPs, and NPs possibly denature the root cell membrane and ultimately promote water and nutrient uptake through membrane stability and nutrient homeostasis [49], increasing the plant biomass. Consistent with our findings, γ-Fe_2_O_3_ and Fe_3_O_4_ application improve the growth, weight, and vitamin contents of the watermelon [50]. The application of γ-Fe_2_O_3_ and Fe_3_O_4_ significantly improves the germination rate, plant biomass, and pigmentation of barley [33]. As α-Fe_2_O_3_ is less magnetic than γ-Fe_2_O_3_ and Fe_3_O_4_, α-Fe_2_O_3_ may decreases the plant biomass and chlorophyll content [51].

### 3.3. Effect of Fe NPs Supply on Chlorophyll Content

**Figure 2:**
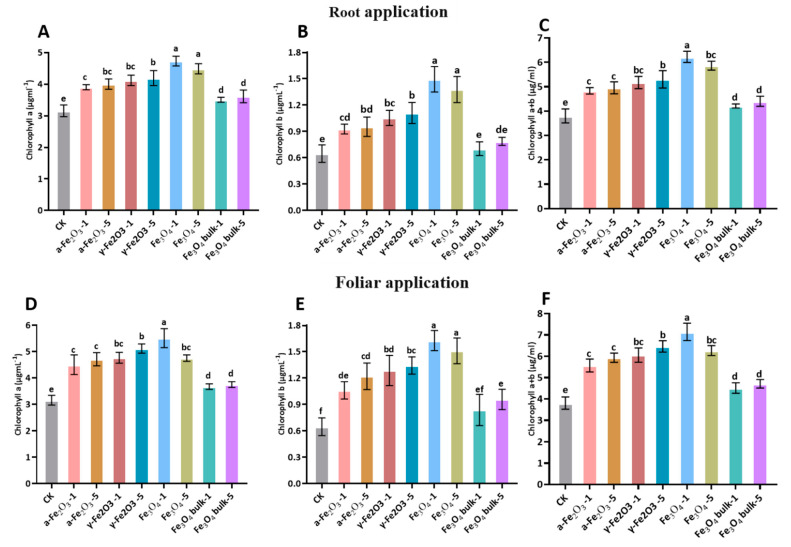
Chlorophyll a, Chlorophyll b, and Total Chlorophyll a + b in maize seedling by root and foliar applied Fe NPs. Root Application: (**A**) Chlorophyll a, (**B**) Chlorophyll b, (**C**) Total Chlorophyll a + b. Foliar application: (**D**) Chlorophyll a, (**E**) Chlorophyll b, (**F**) Total Chlorophyll a + b. The same lowercase letter indicates no significant difference among treatments via Tukey’s HSD at the *p* < 0.05 level.

Iron oxide NPs were impressive in improving the chlorophyll content in maize seedlings (Table 2). Total chlorophyll was found to be maximized by Fe_3_O_4_ application at 500 mg L^−1^, 46% higher than the control, followed by α-Fe_2_O_3_ and γ-Fe_2_O_3_ at 500 mg L^−1,^ which were 41% and 36%, respectively, by foliar application. In the case of root application, chlorophyll a + b was 38% higher than the control treatment by Fe_3_O_4_ (Figure 2C,F). The chlorophyll content increased with increasing concentrations of Fe oxide NPs from 100 mg/L to 500 mg/L (Table 2) with in treatments. Maximal chlorophyll a and chlorophyll b was recorded by Fe_3_O_4_ NPs followed by α-Fe_2_O_3_ and γ-Fe_2_O_3_, having a significant difference with control and other treatments (Figure 2A,B). Foliar application increased the chlorophyll a more than root application but, within treatment, the same trend was observed as in root application (Figure 2D,E). As Fe is the main component in the structure and function of photosynthetic apparatus, Fe increased the chlorophyll content in maize seedlings. According to previous studies, Fe deficiency degrades chloroplast and reduces chlorophyll content [52]. Ref. [53] reported that increasing Fe concentration to 250 mg L^−1^ enhances the chlorophyll content in barley. Similarly, in citrus maxima seedlings, chlorophyll content is significantly enhanced by 23% by foliar application of Fe [54]. Fe oxide NPs translocate from root-to-shoot and increase soybean chlorophyll [30]. Overall, chlorophyll content in maize seedlings leaves depends on the concentration and type of NPs.

### 3.4. Antioxidant Enzyme Activity

**Figure 3:**
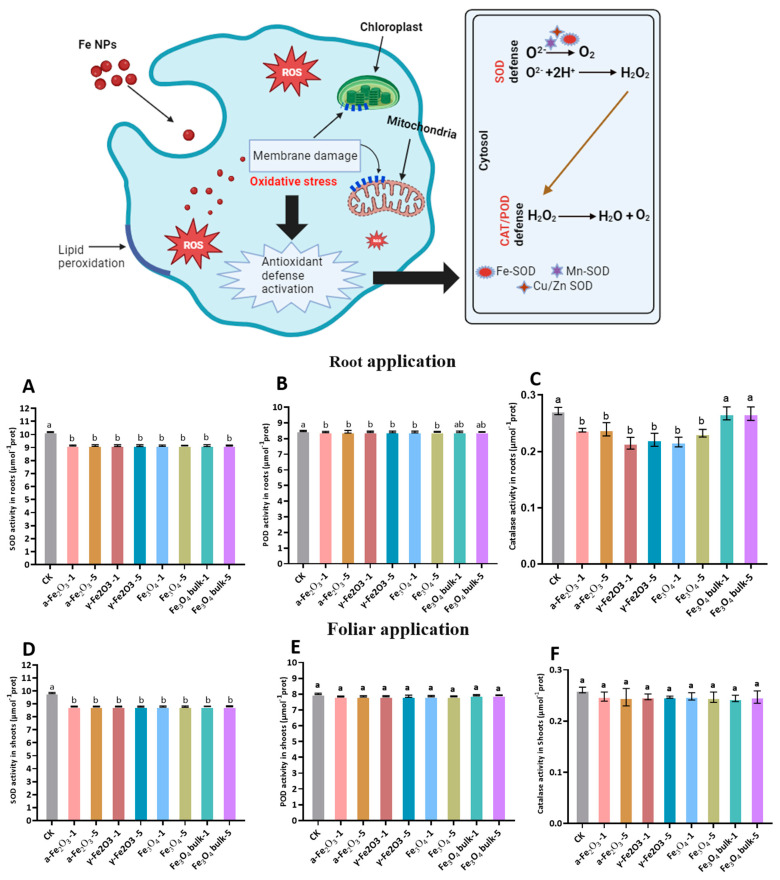
Antioxidants (SOD, POD, and CAT) activities of maize seedling affected by root and foliar application of Fe NPs. Root application: (**A**) SOD activity in roots, (**B**) POD activity in roots, (**C**) CAT activity in roots. Foliar application: (**D**) SOD activity in shoots, (**E**) POD activity in shoot, and (**F**) CAT activity in shoot. The same lowercase letter indicates no significant difference among treatments via Tukey’s HSD at the *p* < 0.05 level.

Reactive oxygen species (ROS) such as hydrogen peroxide (H_2_O_2_) and superoxide (O_2_) can lead to oxidative stress in plants. Plant produces antioxidants such as SOD, POD, and CAT to combat the toxicity of ROS [55]. Figure 3 describes the enzyme activity of maize root and shoot treated with Fe oxide NPs (α-Fe_2_O_3_, γ-Fe_2_O_3_, Fe_3_O_4_, and bulk Fe_3_O_4_) with 100 mg L^−1^ and 500 mg L^−1^. Three types of SOD, namely, Fe-SOD, Mn-SOD, and Cu, Zn-SOD can quickly convert O_2_ to H_2_O_2_ in plant cells. CAT is a well-known antioxidant enzyme that converts H_2_O_2_ to H_2_O and O_2_ [56]. In our study, SOD activity with both soil and foliar applications of Fe NPs was not changed significantly across different treatments, but it was about 12% lower than the control (Figure 3A,D). SOD localizes within the mitochondria and chloroplast by Fe cofactor assembly [57]. Root application of Fe oxide NPs significantly altered the CAT activity of maize seedlings (Figure 3B,E). However, no effects were noted for the foliar application among different treatments. The root application of bulk Fe_2_O_3_ increased the CAT activity, which may be due to the degradation of H_2_O_2_. The no negative response of maize root suggested that oxidative stress did not occur in the root tissues. POD activity of root and shoot was not affected significantly with root and foliar application of Fe oxide NPs (Figure 3C,F). The results of antioxidant enzymes suggested that Fe NPs could activate the defense system in plants when applied through roots. Iron deficiency promotes oxidative stress, but in our study, Fe NPs were applied, which may have fulfilled the Fe nutritional requirements of maize seedlings. Furthermore, antioxidant enzyme activities and ROS generation vary with plant species, size of NPs, and exposure condition [56]. For instance, pumpkin and ryegrass response differently regarding enzymatic activity with exposure to Fe_3_O_4_ NPs [58].

### 3.5. Fe Uptake through Root and Shoot in Maize Seedlings

**Figure 4:**
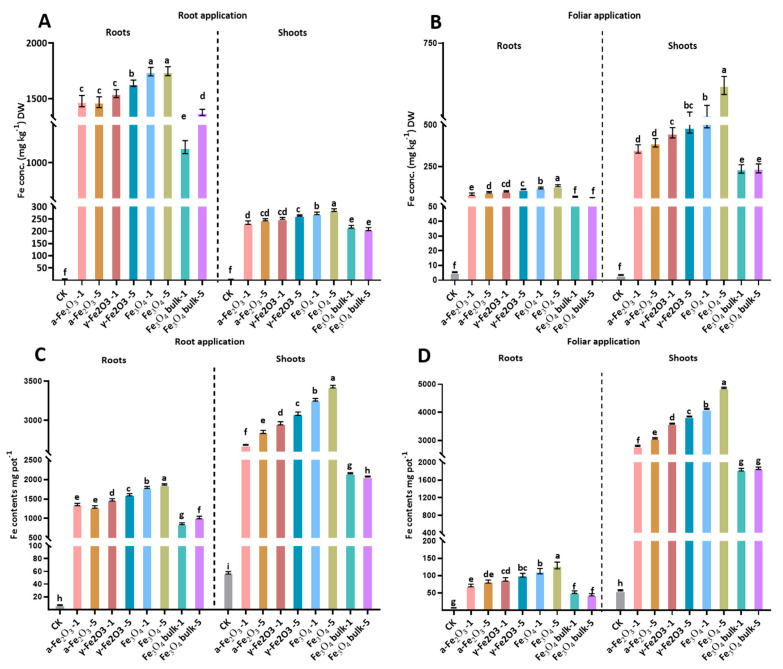
Fe concentration and Fe contents in root and shoot tissues influenced by root and foliar application of different Fe NPs. (**A**) Fe concentration in root and shoot by root application. (**B**) Fe concentration in root and shoots by foliar application. (**C**) Fe contents in root and shoot by root application (**D**) Fe contents in roots and shoot by foliar application. The same lowercase letter indicates no significant difference among treatments via Tukey’s HSD at the *p* < 0.05 level.

**Figure 5:**
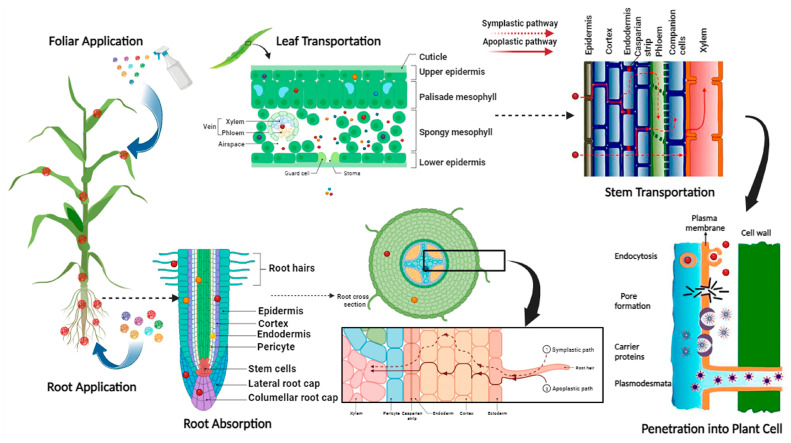
Proposed mechanism of uptake and translocation of root and foliar-applied Fe oxide NPs in maize seedlings (Created by Bio Render.com).

Significant difference in Fe concentration in roots and shoots of maize seedlings was found by Fe NPs types, application methods, and application rate (Table 3). In the case of Fe uptake, mean difference of Fe concentration in the root by two application methods (root vs. foliar) was 1217 and 81 mg kg^−1^ DW. However, mean difference of Fe concentration in shoots (root vs. foliar) was 200 and 330 mg kg^−1^ DW, respectively. These results suggested that maize roots and leaves efficiently absorbed Fe NPs on the surface due to smaller size, surface charge and magnetic characteristics by different Fe oxide sources (α-Fe_2_O_3_, γ-Fe_2_O_3_, Fe_3_O_4_, bulk Fe_3_O_4_). By comparing treatments with control in root application following the trend in root and shoot Fe concentration was observed Fe_3_O_4_ > γ-Fe_2_O_3_ > α-Fe_2_O_3_ > bulk Fe_3_O_4_ (Figure 4A). With foliar application of Fe oxide NPs, Fe_3_O_4_ NPs at the rate of 500 mg L^−1^ showed maximum intake by the shoot of maize seedlings, followed by Fe_3_O_4_ NPs at 100 mg L^−1^, γ-Fe_2_O_3_, α-Fe_2_O_3_ and then bulk (Figure 4B). Overall, Fe concentration in maize plants increased significantly with increasing Fe application rate (Table 3). Iron contents in maize plants with root application of NPs were significantly higher than that in plants with foliar application. Surprisingly, Fe contents in the shoot were higher with root application (Figure 4C). We speculated that it was due to the higher shoot DW with root application of Fe_3_O_4,_ γ-Fe_2_O_3_, and α-Fe_2_O_3_. In contrast, by foliar application, Fe contents were greater in shoots than roots (Figure 4D), which would be due to the greater amount of Fe left in the leaves through the foliar spray of Fe NPs. Iron contents were significantly improved by increasing the input concentration of NPs treatments by both application methods. The plant response to NPs can vary based on the physical (i.e., size, shape) as results showed in our findings and chemical (i.e., surface charge, chemical composition, and surface modification) of applied NPs. For instance, the foliar application of α-Fe_2_O_3_ with a size range (22.3–67.0 nm) an average of 40.9 nm reduces its uptake efficiency in *Arabidopsis* [51], and α-Fe_2_O_3_ with a particle size of 14 nm efficiently utilized by barley [39]. Microscopic evidence shows that hematite and ferihydrite translocate in maize seedlings by 76% and 127%, compared to the control [59]. Notably, the magnetic properties of γ-Fe_2_O_3_ and Fe_3_O_4_ NPs also support the uptake of NPs. The size of aggregated NPs could be larger than the pores space of the cell wall, except for a few that freely translocate through the endodermis and cortex [60]. Fe NPs enter leaves either by a cuticle or stomata pathways, whereas in roots they enter through apoplastic and symplastic pathways (Figure 5). We used NPs of 10–30 nm size, and the maximum size of NPs that can enter the stomata 10–100 μm [61]. As maize is a monocotyledon, adsorption of NPs from leaves is lower in monocots than in the root because of the lesser number of stomata than dicots [62]. Importantly, NPs can stick with organic acids and ligands on the leaf surface, which increase the exposure time of NPs [61]. Thus, smaller sizes with more surface area give an extra advantage to NPs to adsorb on the leaf surface with organic acids and other metabolites [63]. The epidermis composition and function of the root is similar to leaves but the tip of the plant root and root hair surface might not fully develop the epidermis, which possibly helps NPs to enter the root column [64]. However, direct evidence of NPs’ mobility in plants is still unclear. Therefore, assessing the absorption and transport mechanism(s) of NPs in plants is critical to establish adequate nano-enabled agriculture [61].

### 3.6. Confocal Laser Scanning Microscopy

**Figure 6:**
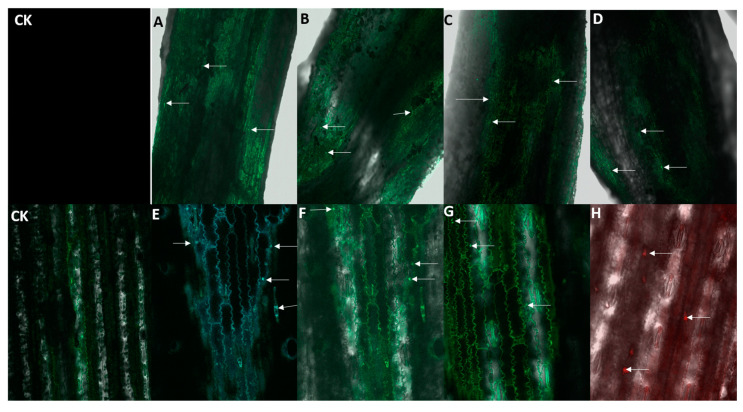
Confocal laser scanning microscopy of root and leave by root and foliar application of Fe oxide NPs. Root: CK, (**A**) Fe_3_O_4_, (**B**) γ-Fe_2_O_3_, (**C**) α-Fe_2_O_3_, (**D**) bulk Fe_3_O_4_. Shoot: CK, (**E**) Fe_3_O_4_, (**F**) γ-Fe_2_O_3_, (**G**) α-Fe_2_O_3_, (**H**) bulk Fe_3_O_4_.

Finally, to gain better insight into Fe oxide NPs translocation in maize seedlings, root and leaves anatomy was observed using the confocal laser scanning microscopy (CLSM) (Figure 6). CLSM results showed the surface absorption of Fe oxide NPs on the epidermal layer of maize root. Fluorescent Fe oxide NPs enter by denaturing the surface of the epidermis to the cortex, and then to the endodermis (Figure 6A–D). Additionally, Fe oxide NPs containing higher magnetism property were adsorbed more on the root surface, which was largely due to their specific surface charge. Iron nanoparticles get entered into leaves through stomata and the cuticle layer shown in Figure 6E–H. Similarly, Zinc nanoparticles were detected through CLSM in sugarcane roots. Zinc nanoparticles were also attached more on the surface of the epidermis and found in the endodermis and cortex [47]. In pumpkin, carbon-coated Fe NPs were detected via CLSM inside the cortex cell located next to the internal hollow of the petiole [65]. Foliar-applied liposome NPs loaded with Fe and Mg were translocated from shoot-to-root and adjacent leaves in cherry tomato. The presence of NPs in root and adjacent leaves was confirmed by CSLM [66].

## 4. Conclusions

In this study, we investigated the comparative effects of root and foliar application of four Fe oxide NPs (α-Fe_2_O_3_, γ-Fe_2_O_3_, Fe_3_O_4_, and bulk Fe_3_O_4_) on the growth and Fe nutrition of maize seedlings. We concluded that: (i) Fe oxide (α-Fe_2_O_3_, γ-Fe_2_O_3_, Fe_3_O_4_ NPs, and bulk Fe_3_O_4_) has a positive impact on maize seedling growth and Fe nutrition; (ii) the application of Fe oxide NPs to the roots is more effective than foliar application in improving Fe concentration, root length, plant biomass, and chlorophyll content. This may be attributed to the translocation of Fe oxide NPs in the shoot, as confirmed by confocal microscopy and the longer exposure time of NPs to the roots; and (iii) overall, Fe_3_O_4_ NPs are the most efficient NPs among the four tested Fe sources, followed by γ-Fe_2_O_3_ and α-Fe_2_O_3_, when applied at a rate of 100 mg L^−1^. In a nutshell, the results of this study suggest that applying Fe_3_O_4_ directly to the roots at a concentration of 100 mg L^−1^ can serve as a promising Fe fertilizer to enhance maize yield. In future studies, the efficiency of α-Fe_2_O_3_, γ-Fe_2_O_3_, and Fe_3_O_4_ NPs regulated by site-specific covariates (i.e., soil properties and climatic conditions) should be investigated in detail and potential environmental risk with bioaccumulation of Fe NPs in food chain can be demonstrated in detail. The industrial application of Fe nanoparticles as absorbent of heavy metals from wastewater and drug delivery in nanomedicine is an interesting topic to be discussed in future studies.

## Figures and Tables

**Table 1:** Root length, shoot height, and dry weight affected by root and foliar application of different Fe oxide NPs.

Treatments	Root Application				Foliar Application			
	Shoot DW (mg pot^−1^)	Shoot Height (cm)	Root DW (mg pot^−1^)	Root Length (cm)	Shoot DW (mg pot^−1^)	Soot Height (cm)	Root DW (mg pot^−1^)	Root Length (cm)
Control	7.6 ± 0.3 c	58.5 ± 0.3 c	0.5 ± 0.02 f	30.3 ± 0.3 d	7.6 ± 0.02 d	58.5 ± 0.3 c	0.5 ± 0.02 e	30.3 ± 0.3 ab
α-Fe_2_O_3_-1	11.4 ± 1.8 a	86.5 ± 0.9 a	0.9 ± 0.04 cd	47.5 ± 1.8 bc	7.8 ± 0.04 bc	74.8 ± 1.8 ab	0.6 ± 0.01 c	32.5 ± 0.9 a
α-Fe_2_O_3_-5	11.5 ± 0.9 a	86.6 ± 0.7 a	0.8 ± 0.03 d	49.7 ± 0.9 bc	7.8 ± 0.03 bc	75.0 ± 0.9 ab	0.6 ± 0.02 c	32.6 ± 0.7 a
γ-Fe_2_O_3_-1	11.7 ± 1.7 a	86.8 ± 0.1 a	0.9 ± 0.03 bd	50.7 ± 1.7 bc	7.9 ± 0.03 bc	75.9 ± 1.7 a	0.6 ± 0.04 bc	32.8 ± 0.1 a
γ-Fe_2_O_3_-5	11.6 ± 1.5 a	86.6 ± 0.3 a	0.9 ± 0.02 bc	51.7 ± 1.5 b	7.9 ± 0.02 abc	75.8 ± 1.5 a	0.6 ± 0.02 bc	32.7 ± 0.3 a
Fe_3_O_4_-1	11.9 ± 1.3 a	86.5 ± 0.4 a	1.0 ± 0.04 ab	57.3 ± 1.3 a	7.9 ± 0.04 ab	76.5 ± 1.3 a	0.7 ± 0.06 ab	32.9 ± 0.4 a
Fe_3_O_4_-5	11.9 ± 2.0 a	87.6 ± 0.3 a	1.0 ± 0.04 a	57.8 ± 2.0 a	8.0 ± 0.04 a	76.6 ± 2.0 a	0.7 ± 0.08 a	33.2 ± 0.3 a
Bulk Fe_3_O_4_-1	9.8 ± 1.1 b	80.8 ± 1.0 b	0.7 ± 0.02 e	41.6 ± 1.1 bc	7.7 ± 0.02 cd	70.6 ± 1.1 b	0.5 ± 0.03 d	28.8 ± 1.0 ab
Bulk Fe_3_O_4_-5	9.9 ± 1.7 b	81.1 ± 0.3 b	0.7 ± 0.04 e	44.2 ± 1.7 c	7.7 ± 0.04 cd	70.5 ± 1.7 b	0.5 ± 0.04 d	28.1 ± 0.3 ab

Mean with same letters in column are not significantly different at (*p* < 0.05) Tukey’s HSD.

**Table 2:** Results of three factor ANOVA (*p*-values) for root, shoot dry weight, root length, and shoot height, chlorophyll (Chl) content of maize seedling.

Sources	DF	Root DW	Shoot DW	Root Length	Shoot Height	Chl a	Chl b	Chl a + b
Fe NPs type (T)	4	0.0000	0.0000	0.0000	0.0000	0.0000	0.0000	0.0000
NPs rate (R)	1	0.3762	0.9307	0.2941	0.8015	0.8492	0.3714	0.7476
Application methods (M)	1	0.0000	0.0000	0.0000	0.0000	0.0000	0.0000	0.0000
T × R	4	0.5825	0.9412	0.9702	0.9947	0.0000	0.1473	0.0001
T × M	4	0.0006	0.0000	0.0000	0.0000	0.0000	0.1496	0.0000
R × M	1	0.5849	0.7066	0.2380	0.8630	0.7097	0.5723	0.9908
T × R × M	4	0.7979	0.9119	0.8697	0.9975	0.0754	0.9436	0.2180

Fe NPs type (T), NPs rate (R), Application methods (M), Degree of Freedom (DF).

**Table 3:** Results of three factor ANOVA (*p*-values) for Fe concentration and content in root and shoot of maize seedling.

Sources	DF	Fe Content in Root	Fe Content in Shoot	Fe Concentration in Root	Fe Concentration in Shoot
Fe NPs type (T)	4	0.0000	0.0000	0.0000	0.0000
NPs rate (R)	1	0.0000	0.0000	0.0000	0.0002
Application methods (M)	1	0.0000	0.0000	0.0000	0.0000
T × R	4	0.0000	0.0000	0.0000	0.0036
T × M	4	0.0000	0.0000	0.0000	0.0000
R × M	1	0.0000	0.0000	0.0002	0.0056
T × R × M	4	0.0000	0.0000	0.0000	0.0885

## Data Availability

Data are contained within the article and Supplementary Materials.

## Supplementary Materials

The following supporting information can be downloaded at: https://www.mdpi.com/article/10.3390/nano13233036/s1, Figure S1. Root morphology of maize seedlings by root and foliar application of Fe oxide NPs.
