# Supplementary material for: Characterization of Root and Foliar-Applied Iron Oxide Nanoparticles (α-Fe2O3, γ-Fe2O3, Fe3O4, and Bulk Fe3O4) in Improving Maize (Zea mays L.) Performance
Source: Nanomaterials (Basel). 2023 Nov 28;13(23):3036. doi: 10.3390/nano13233036 (PMC10708543; doi:10.3390/nano13233036)

Supplementary Figure S1

Figure S1 Root morphology of maize seedlings by root and foliar application of Fe oxide NPs. Root application: CK, (A) Fe_3O_4, (B) γFe_2O_3, (C) α-Fe_2O_3, (D) bulk Fe_3O_4, (E) Fe_3O_4, (F) γFe_2O_3, (G) α-Fe_2O_3, (H) bulk Fe_3O_4. Foliar application: CK, (A) Fe_3O_4, (B) γFe_2O_3, (C) α-Fe_2O_3, (D) bulk Fe_3O_4, (E) Fe_3O_4, (F) γFe_2O_3, (G) α-Fe_2O_3, (H) bulk Fe_3O_4 (A-D) 100 mg L^{-1} (E-F) 500 mg L^{-1}

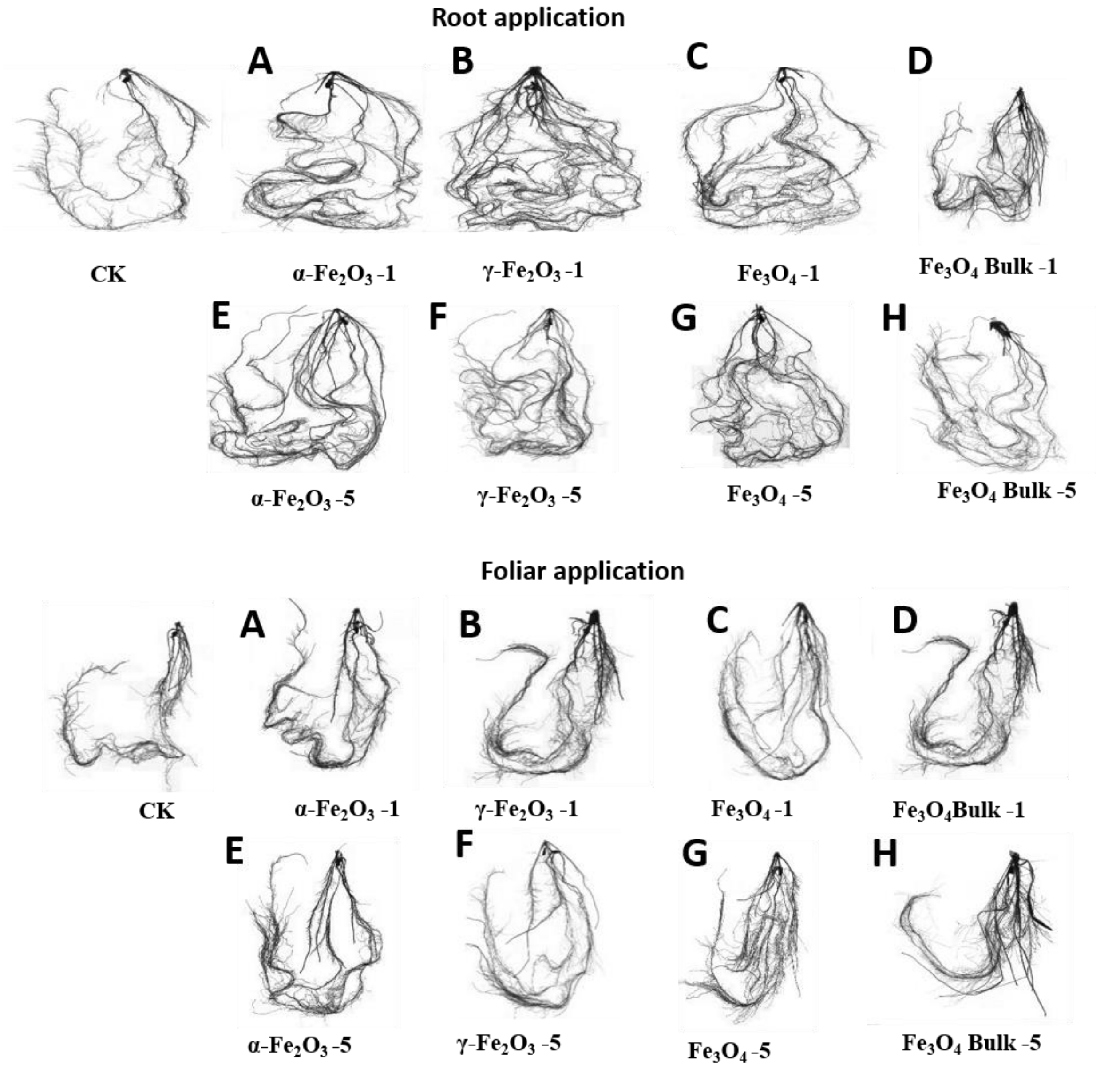

Supplement: Supplementary file 1 [file nanomaterials-13-03036-s001.zip › nanomaterials-2730531-supplementary.pdf]
